# Supplementary material for: Spatial variations and determinants of timely completion of vaccination in Ethiopia using further analysis of EDHS 2019 data: Spatial and multilevel analysis
Source: PLoS One. 2024 Apr 5;19(4):e0301409. doi: 10.1371/journal.pone.0301409 (PMC10997123; doi:10.1371/journal.pone.0301409)
Supplement: Attachment — Submitted filename: respose to reviewers.pdf [file pone.0301409.s002.pdf]

**Spatial variations and determinants of timely completion of vaccination in Ethiopia using further analysis of EDHS 2019 data: Spatial and multilevel analysis.**

Muluken Chanie Agimas*[1], Aysheshim Kassahun Belew[2], Mekonnen Sisay[2], Lemlem Daniel Baffa[2], Moges Gasahw[3], Zufan Yiheyis Abriham[5], Esmael Ali Muhammad[2], Zeamanuel Anteneh Yigzaw[4] Berhanu Mengistu[2]

1 Department of Epidemiology and Biostatistics, institute of public health, College of Medicine and health science, University of Gondar, Gondar, Ethiopia.

Authors address:

2Department of Human Nutrition, Institute of Public Health, College of Medicine and Health Sciences, University of Gondar, Gondar, Ethiopia

3Department of Physiotherapy, School of Medicine, College of Medicine and Health Sciences, University of Gondar, Gondar, Ethiopia

4Department of health promotion and Behavioral Science, school of public health, college of Medicine and health sciences; Bahir Dar University.

5Department of Medical Parasitology, School of Biomedical and Laboratory Sciences, College of Medicine and Health Sciences, University of Gondar, Gondar, Ethiopia

Authors and emails

Muluken Chanie Agimas (MCA): mulukensrc12@gmail.com

Aysheshim kassahun Belew(AKB): aysheshim121@gmail.com

Mekonnen Sisay (MS): mekudesu@gmail.com

Lemlem Daniel Baffa(LDB): lemmidan@gmail.com

Moges Gashaw(MG): mogesgashaw1@gmail.com

Zufan Yiheyis Abriham(ZYA): zufanyiheyis19@gmail.com

Zeamanuel Anteneh Yigzaw(ZAY): zeamanuel19@gmail.com

Esmael Ali Muhammad(EAM): esmaelali34@gmail.com

Berhanu Mengistu (BM): berhanu5@yahoo.com

Corresponding author:

MCA: mulukensrc12@gmail.com

**To: PLOS ONE <em@editorialmanager.com>**

**Manuscript Number: PONE-D-23-12639**

**Cover Letter for reviewer's comments and authors responses**

We would like to thank both the Editorial board members and reviewers for kind comments and suggestions. Accordingly, we have tried to correct and respond to those comments point by point, page by page, and line by line. Therefore, we prepared this author's response, the revised manuscript, the separate revised figures and tables, and the tracked changes of the manuscript.

Sincerely, with best regards!

Muluken Chanie Agimas

ID: https://orcid.org/0000-0003-0808-124X

| Comments from review and editorial board | Authors response |
|---|---|
| Please ensure that your manuscript meets PLOS ONE's style requirements, including those for file naming. The PLOS ONE style templates can be found at | Dear Editor, it is a very interesting comment, and we accepted it so that we can assure you that our manuscript fulfils all the requirements. |
| We note that you have stated that you will provide repository information for your data at acceptance. Should your manuscript be accepted for publication, we will hold it until you provide the relevant accession numbers or DOIs necessary to access your data. If you wish to make changes to your Data Availability statement, please describe these changes in your cover letter and we will update your Data Availability statement to reflect the information you provide. | Great comment. You are right. We stated that we "provide repository information." But we assure you that we provided this answer erroneously. So we would like to change the data availability statement (we will not provide repository information). |

| | |
|---|---|
| Please include a separate caption for each figure in your manuscript. | It is great, and you are right. As I read, PLOS ONE requires a separate caption in the manuscript. Therefore, the necessary amendment has been made accordingly. |
| Please include your tables as part of your main manuscript and remove the individual files. Please note that supplementary tables (should remain/ be uploaded) as separate "supporting information" files. | Thank you, dear editor. You are right, and we did it. |
| We note that Figures 3, 5, 6 & 8 in your submission contain [map/satellite] images which may be copyrighted. All PLOS content is published under the Creative Commons Attribution License (CC BY 4.0), which means that the manuscript, images, and Supporting Information files will be freely available online, and any third party is permitted to access, download, copy, distribute, and use these materials in any way, even commercially, with proper attribution. For these reasons, we cannot publish previously copyrighted maps or satellite images created using proprietary data, such as Google software (Google Maps, Street View, and Earth). For more information, see our copyright guidelines: http://journals.plos.org/plosone/s/licenses-and-copyright.<br><br>We require you to either (1) present written permission from the copyright holder to publish these figures specifically under the CC BY 4.0 license, or (2) remove the figures from your submission: | Dear Editor, you are right; images are freely accessible and easily copied. But deadly sure, the images were never copied from other sources; rather, the image was from ARC GIS output. Within the image, legend and figure titles describe the real output from ARC GIS with real data. If it were copied from another source, the title of the figure and legend would never match our research. The second is that there is no similar study output in Ethiopia entitled to timely vaccination in Ethiopia using EDHS data to be copied from the previous output. But the shape file of Ethiopia was obtained from (CSA 2013). Using this shape file, timely vaccination of EDHS-2019 data was added and analyzed using ARC GIS Version 10.3. Therefore, the source of the shape file is included in the image. The legends and the titles of the figures also provide evidence for not copying |

| | |
|---|---|
| | from another source. Because the legend and the title of the figure are labeled (given a name) only in the Arc GIS software. |
| If any of the maps in Figures 3, 5, 6 & 8 have been previously copyrighted, we require specific consent from the copyright holder(s) to publish these images in PLOS ONE, under the CC BY 4.0 license.<br><br>Please confirm if the data used to create the maps in Figures 3, 5, 6 & 8 is from the DHS | None of the figure is copied from other sources. So written permission from the copyright holder was not applicable. Copyright information for the Ethiopia (CSA 2013) was not obtained. These shape file does not require copyright information. Because shape file of Ethiopia was taken from open source (Volunteer driven open data source).<br><br>Dear editor, our work didn't violate the all listed Conditions of Use for The DHS Program datasets. We request the DHS data to show the distribution and determinants of age appropriate vaccination in Ethiopia using 2019 EDHS data set by taking shape files from CSA 2013, Ethiopia. The map was not obtained from DHS. Rather the shape file of Ethiopia was taken from open source (Volunteer driven open data source platform) |

| Reviewer one comments | Authors response |
|---|---|
| Reviewer #1: The data source and permission is well .stated in the article<br>Line 52 The ANC follow-up was not fully written<br>Line 53 The age cut off was not written<br>Figure 1 The legend " not timely completion of vaccination..." does not read well<br>Figure 2 Measles was misspelled | Dear reviewer It is a very interesting comment. You have seen our manuscript attentively, and we would like to thank you for your scientific contribution to making our manuscript valuable for intervention and policymaking. We accept your valuable comments. So the necessary amendment has been made accordingly. |
| **Reviewer two comments** | **Authors response** |
| The abstract section of your manuscript clearly justifies the study, however, the study could benefit from inclusion of the aim of the study with justification in the introduction section of the abstract instead of separately putting it as objective. It the authors have made it based on the journal guideline please leave this concern. | Great, you are right. If it is written with an introduction, it makes more sense. But the journal recommends that the abstract be written in a separate and structured way. |
| Abstract methods; the authors described about the methodology of the data source instead of describing the authors' own study. This section has incorporated too much information. The authors need to consider that they are in abstract and need to come with only main points of the methodology. For example, use of AIC for model selection is very specific and should be clearly addressed in the method section of body of manuscript with its importance not in the abstract. | Great view! We got our mistake. As you stated, we narrated the previous work. Therefore, revisions have been made precisely in a smart and informative way based on our work. |
| *Under result of your abstract you did not have mentioned anything about results of spatial analysis, which I strongly recommend you to mention it. In general, an abstract of a study should briefly address the burden of the problem, need for study, relevance, aim, method, results and conclusion with recommendation. The study could benefit from modification of the abstract. | Really, I am lucky when you are assigned as a reviewer for our paper. We have learned a lot and gained experience from your comments, even for future manuscript preparation. Without defending your comment, a necessary |

| | |
|---|---|
| | amendment has been made in the abstract section accordingly. |
| The introduction section of this manuscript best describes the background information regarding the problem its burden. Taking that into account, "According to the World Health Organization, timely vaccination is the practice of administering the vaccine within the first birthday of a child. (1)." Please remove the period before the reference. All vaccines are not initiated at the day of birth. On that ground, the authors need to come with more comprehensive definition and recommendation of the "timely completion of vaccination" instead of timely initiation. The manuscript could benefit from modification the introduction with incorporation of its requirements. | Sorry for the use of an ambiguous word. When we say "the first birth day," we mean that the first year (the first celebration of birth day; the first birth day is celebrated at one year) does not mean that it is immediately initiated after birth. Rather, we mean that within a 1-year schedule. So to avoid this vague word, we substitute "within a 1-year schedule." So our study is about whether the child takes the vaccine within the 1-year period (based on the WHO recommended schedule). For example, according to WHO and BCG, the polio-0 vaccine should be taken at birth; polio-1, penta-1, and pcv-1 should be taken at one month or after 4 weeks; and other vaccines continued to take up to 1 year. |
| "The source population was all women of Ethiopia who had a live birth aged 12-35 months earlier in the national survey and women who had live birth aged 12-35 months in the selected enumeration was the study population." What is your base for cutoff points or for your study subjects to fall in the age range of 12-35 months? Perhaps, from the dataset, if that was the case, clearly mention it. Could you replace "live | Dear reviewers, You correctly said. Most of your comments are acceptable without trying to defend them. So child is a more preferable word for the targeted population. |

| | |
|---|---|
| birth" with "baby or child"? Live birth would be more appropriate if you were dealing with subjects immediately after birth. | The reason for using the starting cutoff of 12 months is: first, to classify whether a child has completed all vaccines, the child's age should be at least one year (12 months). Because the timely vaccine schedule is completed for up to one year, So it is impossible to interview those children aged less than 12 months (one year) because these children have not completed their vaccine. We have to select those children who are eligible to be interviewed for the question of whether they take all vaccines on time or not. Timely vaccination means taking all vaccines within the recommended vaccination schedule (within one year). For example, for a child under the age of 5 months, we cannot decide whether to take all vaccines within the recommended period. Because at this age, this child is not illegible to vaccinate against all vaccines. The reason we used the maximum cut-off of 35 months or not included those women who had children aged more than 35 months was to reduce recall bias. The other reason why we use this cut-off is because previously the survey was conducted and interviewed among women who had children aged 12-35 months. |

| | |
|---|---|
| *Page 6, line 114, “Timely completion of vaccination (Yes, No)” needs to be described well about your outcome determination with a standard or fact. For example, how did you categorize or dichotomize “yes” or "no"? | Great view, and thank you for asking to clarify. Now we clearly state the outcome of interest in our manuscript.. |
| * Line 118-119, “current breast feeding, total numbers of live birth, ANC follow-up, numbers of ANC visit and current pregnancy,” Please rewrite the sentence. It does not make sense by its current state. Were “ANC follow-up” and “numbers of ANC visit” different variables. | Okay, it is obvious; it creates confusion. So revisions have been made accordingly. |
| *Multilevel analysis was not well described in the methods section. The authors need to thoroughly describe multilevel analysis and its important parameters reported in the results of the study. For example, what multilevel is? Why did the authors use multilevel instead of ordinary logistic regression and how were parameters (ICC, PCV and AIC) determined? What is the case to use AIC instead of deviance or DIC for model selection? | Dear reviewer: The deviance information criterion (DIC) is a hierarchical modeling generalization of the Akaike information criterion (AIC). It is particularly useful in Bayesian model selection problems where the posterior distributions of the models have been obtained by Markov chain Monte Carlo (MCMC) simulation. Whereas the big advantage of AIC for model selection is that it is automatic whenever we can compute the marginal likelihood, and it produces weights that can be used directly for model-averaging predictions or parameters that have a consistent interpretation across models. DIC is a popular Bayesian analog of AIC or BIC. DIC can only be used to compare models with the same likelihood. So once the assumptions of AIC (or AICc) have been met, the biggest advantage is that our models do not need to be nested for the analysis to |

| | |
|---|---|
| | be valid unlike other single-number measurements of model fit like the likelihood-ratio test.<br><br>We used the mixed-effects binary logistic regression model because of the hierarchical nature of the EDHS data. It is impossible to manage the hierarchical data or show the clustering effect using classical binary logistic regression.<br><br>Dear reviewer, as you have mentioned, we feel that the description of multilevel analysis is shallow. So now we describe well with the parameter formula. |
| "Because the data were secondary or EDHS data ethical clearance was not applicable rather online permission was obtained from DHS international." Your ethical consideration is crystal clear, but reader needs more information about source for your data like URL and you should describe Ethical consideration used by DHS. | Great, and thank you for your excellent view. The necessary revision has been made to be more informative. |
| *Would you please explain whether you used false discovery rate correction while you performed hot spot analysis? If yes, please mention that. If you haven't used that, then would you please justify why you didn't use false discover rate correction (which is very important). If it can't be justified, it will be great if the hot spot analysis could be done again. | Dear reviewer, we did not report everything we did during the multilevel and spatial analysis. Definitely, we used the false discovery rate correction during the hot spot analysis. So we added this vital information to our manuscript. |
| *The DHS displaces the geographic locations of the clusters for maintaining confidentialities of the respondents. For this reason, when there is the possibility of using specific geographic location after an analysis, appropriate buffer is recommended to plot in the map | Okay accepted. Dear reviewer, deadly sure, we took a new lesson and experience from your comments. You are correct; buffering the hot spot |

| | |
|---|---|
| (reference: Burgert CR, Prosnitz D. Linking DHS household and SPA facility surveys: Data considerations and geospatial methods. Rockville, Maryland, USA: ICF International; 2014. Report No.: DHS Spatial Analysis Reports No. 10.). I think using an appropriate buffer in hot spot analysis is very important. Is it possible to take an appropriate buffer around only the statistically significant hot and cold spots (cluster)? Otherwise, readers will not be able to understand the area of hot and cold spots. | analysis makes it more informative and understandable for the reader. So the necessary amendment has been made. |
| I found your discussion consistent with your results and rigorous. The results of the study were addressed with rigorous discussion. However, the study could benefit from limitation of spatial analysis in addition to the limitations addressed in the discussion. | Definitely, we can add spatial analysis-related limitations, and an amendment or revision has been made. |

**Thank you, dear editor and reviewers, for your great scientific contribution!**
